# Phytobezoar in a jejunal diverticulum as a cause of small bowel obstruction: a case report

**DOI:** 10.1186/1752-1947-5-482

**Published:** 2011-09-27

**Authors:** Mohammad Tayeb, Faiz Mohammad Khan, Fozia Rauf, M Mumtaz Khan

**Affiliations:** 1 Department of Surgery, Peshawar Medical College, Peshawar Medical College Warsak Road, Peshawar KPK, Pakistan; 2 Department of Pathology, Peshawar Medical College, Peshawar Medical College Warsak Road, Peshawar KPK, Pakistan

## Abstract

**Introduction:**

Phytobezoars are concretions of poorly digested fruit and vegetable fibers found in the alimentary tract. Previous gastric resection, gastrojejunostomy, or pyloroplasty predispose people to bezoar formation. Small-bowel bezoars normally come from the stomach, and primary small-bowel bezoars are very rare. They are seen only in patients with underlying small-bowel diseases such as diverticula, strictures, or tumors. Primary small-bowel bezoars almost always present as intestinal obstructions, although it is a very rare cause, being responsible for less than 3% of all small-bowel obstructions in one series. Jejunal diverticula are rare, with an incidence of less than 0.5%. They are usually asymptomatic pseudodiverticula of pulsion type, and complications are reported in 10% to 30% of patients. A phytobezoar in a jejunal diverticulum is an extremely rare presentation.

**Case presentation:**

A 78-year-old Pakistani man presented to our clinic with small-bowel obstruction. Upon exploration, we found a primary small-bowel bezoar originating in a jejunal diverticulum and causing jejunal obstruction. Resection and anastomosis of the jejunal segment harboring the diverticulum was performed, and our patient had an uneventful recovery.

**Conclusion:**

Primary small-bowel bezoars are very rare but must be kept in mind as a possible cause of small-bowel obstruction.

## Introduction

Phytobezoars are concretions of poorly digested fruit and vegetable fibers found in the alimentary tract [1]. Previous gastric resection, gastrojejunostomy, pyloroplasty, ingestion of high-fiber foods, persimmon fruit ingestion, incomplete mastication habits, and autonomic neuropathies in patients with diabetes are predisposing factors for bezoar formation [2].

Different types of phytobezoars have been reported in the literature. The most common type is the diospyrobezoar, which occurs as a result of the ingestion of persimmons [3]. Pharmacobezoars caused by medicine, shellac bezoars in furniture workers, lactobezoars in neonates, and trichobezoars in psychiatric patients or young girls are other types of bezoars [3,4].

Small-bowel bezoars normally come from stomach, and primary small-bowel bezoars are very rare. They are seen only in patients with underlying small-bowel disease such as diverticula, strictures, or tumors [5]. Primary small-bowel bezoars almost always present as intestinal obstructions, although they are a very rare cause, being responsible for less than 3% of all small-bowel obstructions in one series [6].

Jejunal diverticula are rare, with an incidence of less than 0.5% [7]. They are usually pseudodiverticula of pulsion type, comprised of only mucosa and submucosa arising from the mesenteric border at vascular entry sites. Despite the fact that most patients with jejunal diverticulosis remain completely asymptomatic, complications are reported in 10% to 30% of patients [8-10]. These include chronic abdominal pain, malabsorption, hemorrhage, diverticulitis, bowel obstruction, abscess formation, and, rarely, diverticular perforation [11]. Phytobezoar in a jejunal diverticulum is an extremely rare presentation.

## Case presentation

A 78-year-old Pakistani man was brought to our clinic with a three-day history of epigastric and central abdominal pain associated with vomiting and abdominal distension. He had no history of any loss of weight or appetite. He had no significant medical or surgical history, except for recurrent, vague upper abdominal pain for the past few years that was treated as pain related to dyspepsia.

**Figure 1:**
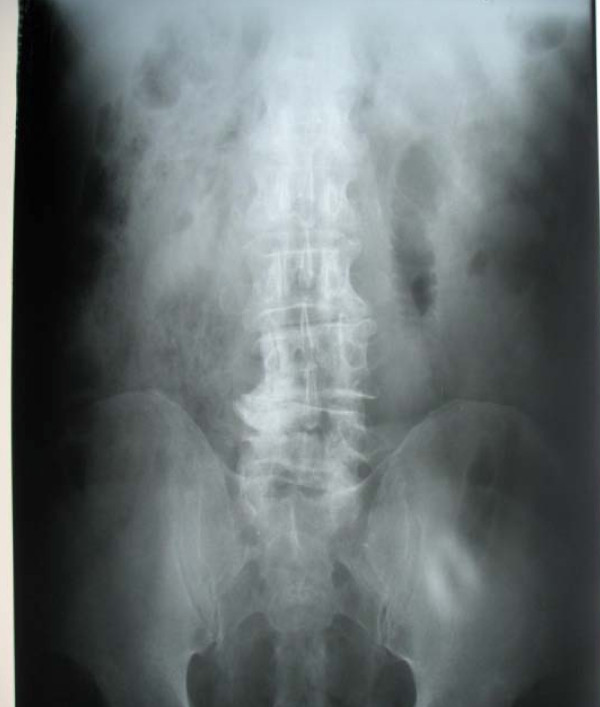
**Supine abdominal X-ray showing dilated jejunal loop on the left side**.

**Figure 2:**
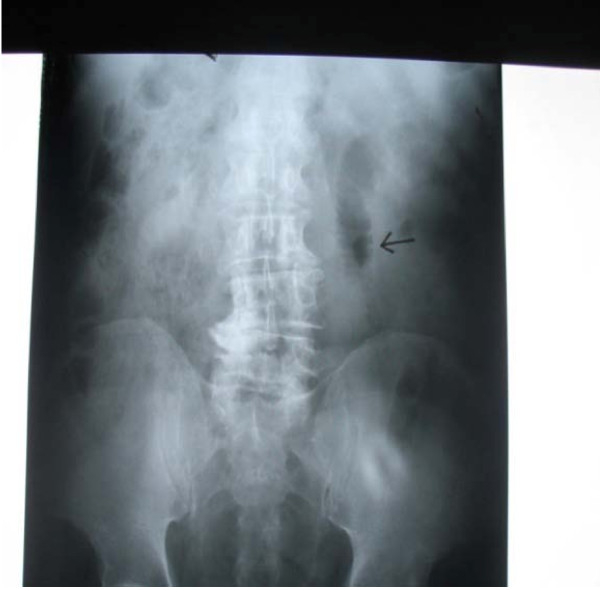
**Supine abdominal X-ray with arrow indicating dilated jejunal loop on the left side**.

Upon admission, his blood pressure was 130/90 mmHg, his pulse was 100 beats/minute, and he was afebrile. An abdominal examination revealed fullness in the epigastrium and a vague, mobile mass with mild tenderness was palpable in the right hypochondrium. His bowel sounds were sluggish, and rectal examination revealed an empty rectum with no palpable mass. His laboratory investigation results were unremarkable. A supine abdominal X-ray (Figure 1 and 2) showed a dilated jejunal loop in the left hypochondrium. Sonography of the palpable mass was suggestive of a possible abscess cavity with internal echoes.

**Figure 3:**
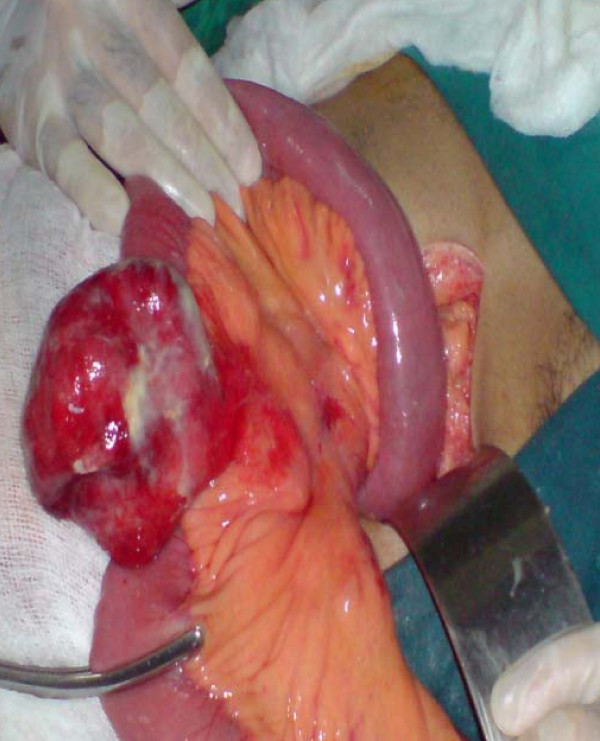
**Proximal dilated jejunal loop with inflamed diverticulum**.

**Figure 4:**
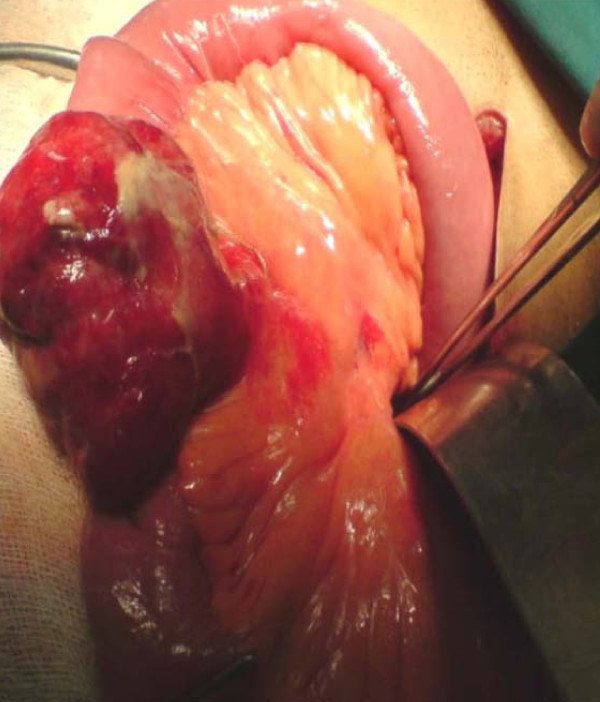
**Proximal dilated jejunal loop with inflamed diverticulum**. The forceps are pointing toward the duodenojejunal junction.

**Figure 5:**
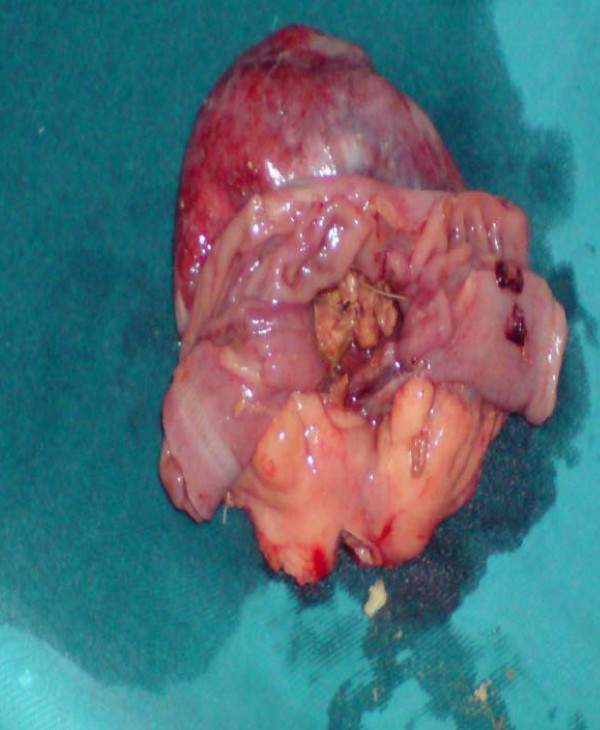
**Specimen opened along the mesenteric border of the bowel**. The phytobezoar can be seen inside the diverticulum.

**Figure 6:**
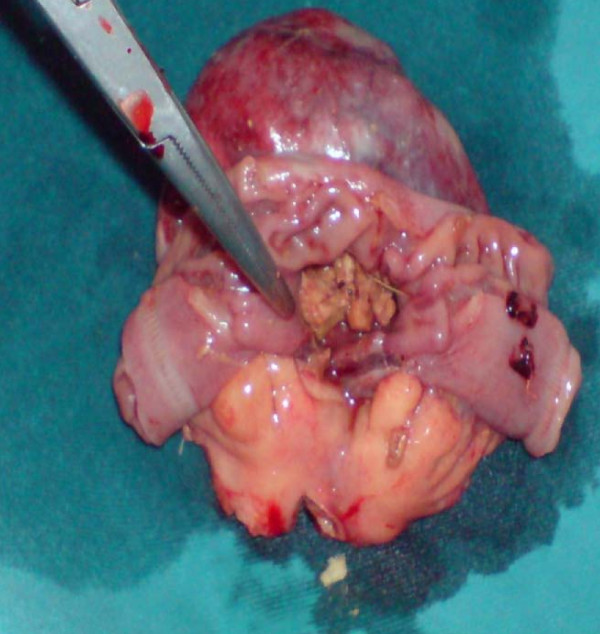
**Phytobezoar can be seen inside the diverticulum (pointing forceps)**.

**Figure 7:**
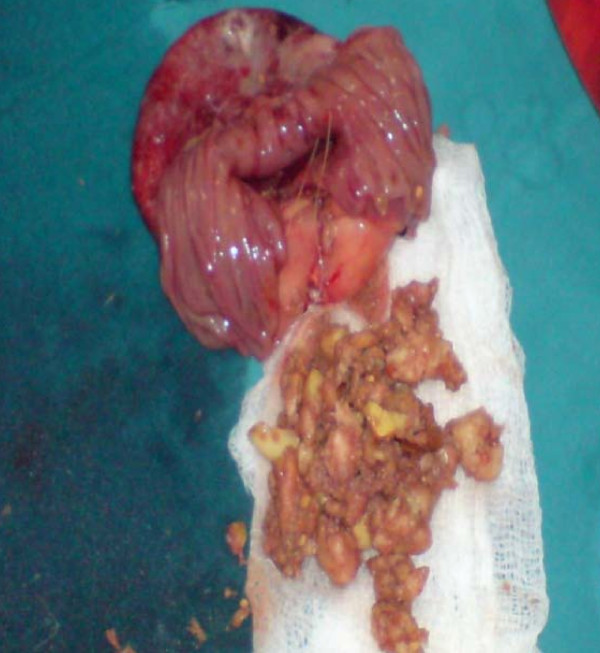
**Phytobezoar, undigested apple pieces, and whole maize grains removed from the diverticulum**.

**Figure 8:**
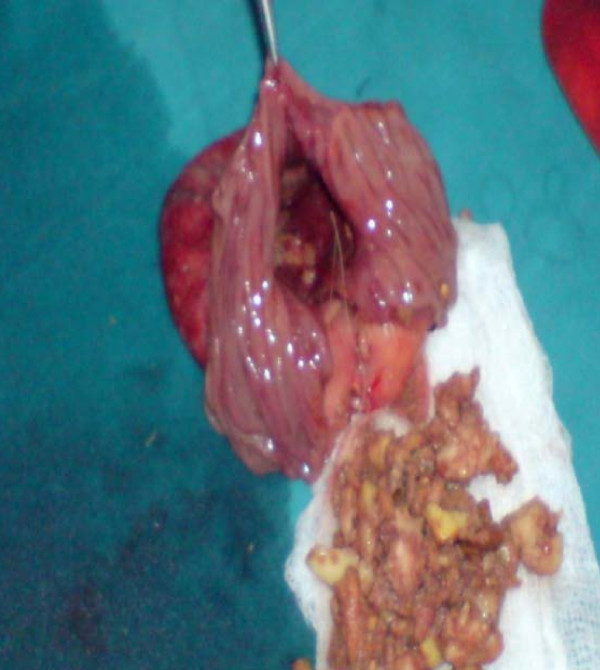
**Empty lumen of the diverticulum**.

On the basis of the clinical and radiological findings, a diagnosis of small-bowel obstruction was made. After resuscitation of the patient, an exploratory laparotomy was performed with the interesting finding of a 7 cm ×5 cm jejunal diverticulum arising from the antimesenteric border of the jejunum about 25 cm distal from the duodenojejunal junction (Figure 3 and 4). The diverticulum was inflamed and full of semi-solid material which was later found to consist of undigested apple pieces with whole maize grains forming a phytobezoar (Figure 5, Figure 6, Figure 7 and 8. Because of the weight of the diverticulum, the jejunum had twisted at its mesentery, causing obstruction. The omentum was wrapped around the diverticulum, forming a phlegmon. Resection and end-to-end anastomosis of the jejunum were performed. The stomach and the rest of the small-bowel examination revealed no other synchronous phytobezoar.

Post-operatively, we noticed that the patient had near total loss of dentition. In response to our inquiry about his dietary habits, he said that he was a fast eater and did not chew food properly, the characteristic feature of an abdominal masticator. The patient had an uneventful recovery.

Histopathology showed a 7 cm × 5 cm × 3 cm diverticulum arising from the antimesenteric border of the jejunum with marked inflammation and necrosis of the mucosa. This was a true diverticulum containing all the layers. There was no evidence of any malignancy or any ectopic gastric or pancreatic mucosa in the diverticulum. Upon his discharge from the hospital, he was counseled about dietary habits and proper mastication to avoid future problems.

## Discussion

The origin of word "bezoar" derives either from the Arabic term "badzehr" or the Persian word "padzahr," both of which denote counterpoison or antidote. This word was applied to a greenish, hard concretion found in the fourth stomach of the Syrian goat. The stone was felt to prevent poisoning and came to Europe as the bezoar stone, which was highly prized for its medicinal properties [12,13].

In our patient, abnormal mastication habits as well as a jejunal diverticulum were the precipitating factors for phytobezoar formation that led to small-bowel obstruction. Although the commonest cause is previous gastric surgery, in our patient there was no such history. Phytobezoars usually occur as single entities, but multiple phytobezoars have been reported in the stomach in 17% of patients and in the intestine in 4% of patients [3,14].

Krausz *et al*. [15] reported a huge increase in the incidence of phytobezoar obstruction in Israel, which was related to the increasing availability and popularity of the persimmon fruit. In Hong Kong, the mid-autumn festival is celebrated in October. During this festival, it is a traditional to eat persimmon fruit. Chisholm *et al*. [16] reported that two-thirds of the patients in their series presented around this festival time.

Common causes of small-bowel obstruction are adhesions, strangulated hernia, malignancy, volvulus, and inflammatory bowel disease. Phytobezoars are rare, accounting for only 0.4% to 4% of all intestinal obstruction. No particular age or sex prevalence has been observed [17]. Primary small-bowel bezoars almost always present as intestinal obstructions.

A number of diagnostic modalities have been used for the detection of abdominal bezoars. Rippolés *et al*. [18] reported a phytobezoar detection rate of 88% by ultrasound in patients with small-bowel obstruction. The main limitation of ultrasound is that it is operator-dependent and may be unreliable, as seen in our case. Computed tomography shows the phytobezoars as a mass, a filling defect, or a fecal ball sign [19], which is considered an accurate diagnostic sign in the preoperative diagnosis of phytobezoar.

Small-bowel bezoars are usually treated surgically. It is mandatory to explore the whole gastrointestinal tract to avoid synchronous bezoars and the recurrence of intestinal obstruction due to retained bezoars. Other described treatment options include enzymatic breakdown and endoscopic fragmentation for gastric bezoars [1,20].

In summary, we have described a case of an elderly man with a small-bowel obstruction due to a phytobezoar complicating a jejunal diverticulum. He had a combination of two rarities, that is, jejunal diverticulum and a phytobezoar as a cause of small-bowel obstruction.

A search of the surgical literature revealed only one case of an obstructing phytobezoar arising from a proximal jejunal diverticulum [21].

## Conclusion

Primary small-bowel bezoars are very rare but must be kept in mind as a possible cause of small-bowel obstruction.

## Competing interests

The authors declare that they have no competing interests.

## Consent

Written informed consent was obtained from the patient for publication of this case report and any accompanying images. A copy of the written consent (in Urdu) is available for review by the Editor-in-Chief of this journal.

## Authors' contributions

MT performed the surgery and wrote the main part of the manuscript. FK reviewed the manuscript and made valuable changes. FR performed the histology in this case and also wrote the pathology part of the manuscript. MK performed the histology and revised the manuscript.

All authors read and approved the final manuscript.
